# Maximizing the Survival of Amyotrophic Lateral Sclerosis Patients: Current Perspectives

**DOI:** 10.1155/2018/6534150

**Published:** 2018-08-12

**Authors:** Osama A. Khairoalsindi, Ahmad R. Abuzinadah

**Affiliations:** ^1^Umm Al-Qura University, College of Medicine, Makkah, Saudi Arabia; ^2^King Abdulaziz University, Internal Medicine Department, Neurology Division, Jeddah, Saudi Arabia

## Abstract

Amyotrophic lateral sclerosis is a neurodegenerative disease that leads to loss of the upper and lower motor neurons. Almost 90% of all cases occur in the sporadic form, with the rest occurring in the familial form. The disease has a poor prognosis, with only two disease-modifying drugs approved by the United States Food and Drug Administration (FDA). The approved drugs for the disease have very limited survival benefits. Edaravone is a new FDA-approved medication that may slow the disease progression by 33% in a selected subgroup of ALS patients. This paper covers the various interventions that may provide survival benefits, such as early diagnosis, medications, gene therapy, stem cell therapy, diet, nutritional supplements, multidisciplinary clinics, and mechanical invasive and noninvasive ventilation. The recent data on masitinib, the role of enteral feeding, gene therapy, and stem cell therapy is discussed.

## 1. Introduction

Amyotrophic lateral sclerosis (ALS) is one of the neurodegenerative diseases that is characterized by a progressive loss of the upper and lower motor neurons at the motor cortex, spinal, and bulbar levels. It is also known as Charcot disease as it was first described by the French neurologist Jean-Martin Charcot [1, 2]. Common initial symptoms include muscle twitching, dysarthria, dysphagia, and localized asymmetric muscle weakness of the upper and lower limbs that progress in myotomal distribution. The disease ultimately leads to atrophy and impairment of the limb muscles [1, 2]. A proportion of patients progress toward bulbar symptoms, which mainly include dysphagia, ventilatory compromise, and sialorrhea [1]. Others are also prone to the pseudobulbar affect (PBA) [1, 3]. The Escorial criteria are commonly used for obtaining a definitive diagnosis of ALS [4, 5]. The underlying etiology is not known. Several genetic mutations have been found to be associated with familial ALS (FALS) which accounts for 10% of ALS [6, 7]. Open reading frame on chromosome 9 (C9Orf72) may account for 35% to 40% of FALS. Other group of genes have been found to be associated with FALS with different mechanism of action such as senataxin (SETX) and fused in sarcoma (FUS) genes which have roles in RNA processing and alsin gene which is involved in endosomal trafficking and valosin containing protein (VCP) which is involved in protein degradation [7]. The accumulating evidence suggests that a mutant part of the antioxidant enzyme superoxide dismutase 1 gene (SOD1) plays a key role in developing FALS and provides insight regarding the pathway causing sporadic ALS (SALS) [8–10]. It is proposed that the mutant SOD1 culminates in developing the disease through glutamate excitotoxicity, mitochondrial dysfunction, oxidative stress induced by free radicals, and impaired axonal transportation [11–14]. The misfolded SOD1 protein was found to be able to spread in a prion-like mechanism which plays role in developing ALS in both FALS and SALS [15]. There are two possible mechanisms of spreading of SOD1 mutant protein [16]. The first mechanism is through uptaking the protein from dying neuron through macropinocytosis. The second mechanism is through uptaking the protein through exosomes of neighboring cells. In the United States, the overall prevalence of ALS in 2013 was five cases per 100,000 persons [17]. Of all the cases of ALS, 90–95% of all cases are SALS, with the remaining 5–10% being transmitted in a genetically inherited manner (i.e., FALS) [14, 18].

Despite the fact that ALS is an incurable disease to date, it is a treatable one but with only modest survival benefits given the scope of currently approved therapeutic strategies. A previous study elucidated that, following symptom onset, less than half of patients survived the first three years [19–22]. As there are no currently addressed medications that can reverse the resultant degeneration, the contemporary aim of management is to alleviate the related symptoms and to ameliorate the progressive degeneration, as well as to attain maximum possible survival. This paper outlines the current perspectives of modifiable factors that may confer a survival benefit upon patients, along with their corresponding evidence, starting from the asymptomatic phase of the disease until the palliative caring phase (Table 1).

## 2. Role of Early Diagnosis Using Genetic Screening

FALS is caused by many specific genetic mutations [13, 23]. Some cases of FALS follow the conventional Mendelian pattern and, thus, genetic counseling for the relatives of patients constitutes an important step in detecting any future possibility of developing the disease [14, 24]. The most relevant gene is C9orf72, which is found in 40% of FALS. C9orf72 is also linked to other neurodegenerative disorders such as frontotemporal dementia, parkinsonism, and Alzheimer's disease. Other common mutations are SOD1 (20%), FUS, and TARDBP genes (1–5% each) [13, 25–27].

There are many at-risk subjects who request genetic testing when they discover a relative diagnosed with ALS. The risk of developing ALS in sporadic ALS relatives is 0.5% for siblings and 1% for offspring [28, 29].

The European Federation of Neurological Societies (EFNS) states in its issued guidelines that “asymptomatic at-risk genetic testing should only be performed in first degree adult blood relatives of patients with a known gene mutation, on a strictly voluntary basis and following accepted ethical principles” [30]. To date, there is no available evidence demonstrating whether the detection of the early asymptomatic phase can prolong survival by any means. Because there are no current preventive or curative agents for the disease, genetic testing is rendered deniable by private insurance companies in the USA [28]. Another addressed reason for not recommending genetic testing routinely is that the molecular tests that detect the mutations of the implicated genes have low predictivities and, as a result, there is unavoidable doubt when conveying the probabilistic risk to the relatives of patients [28].

Despite the nonexistence of agents that can halt the possible development of the disease when it is suspected, genetic testing should be discussed with already-diagnosed ALS patients who have first- or second-degree relatives with ALS, frontotemporal dementia, or both. For the purposes of research studies, other ALS patients should also be offered genetic testing. It also must be emphasized that there are major uncertainties in the interpretation of the test's results.

Aside from the warranted acquisition of knowledge when performing these research studies, however, according to the experiences of Michael Benatar et al., at-risk patients may want to discover whether they have the mutant genes so that they have a sufficient opportunity to make lifelong decisions, e.g., electing not to conceive children for fear of transmitting mutations [31].

## 3. Treatment

### 3.1. Riluzole

**Figure 1:**
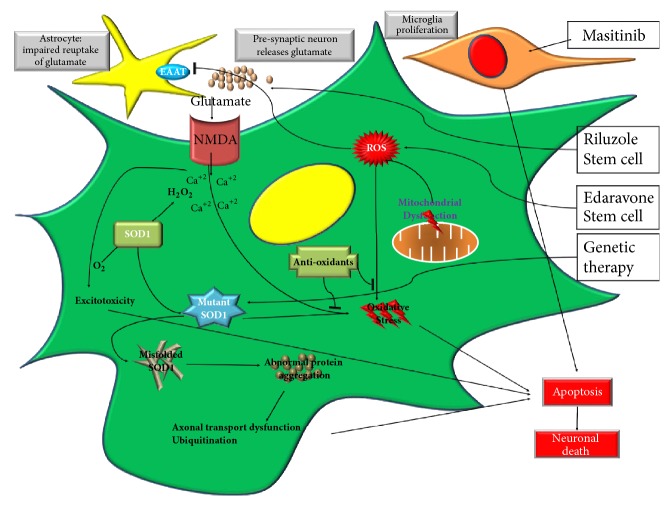
*Signaling Pathway in ALS and Mechanism of Action of Current Therapies*. The signaling pathway demonstrates that the neuronal cell death that occurs in ALS is triggered by several mechanisms and different therapy may target different mechanisms. Glutamate toxicity is targeted by Riluzole. Oxidative stress is targeted by Edaravone and mircoglia induced inflammation is targeted by masitinib. Stem cell therapy may target many of the above mechanisms. ROS: reactive oxygen species. EAAT2: excitatory amino acid transporter 2. NMDA: N*‑*methyl*‑*d-aspartate. SOD1: superoxide dismutase 1.

Riluzole is the first drug to be approved for ALS by the US Food and Drug Administration (FDA). The mechanism of action of riluzole involves the modulation of glutamatergic neurotransmission in the motor cortex and spinal cord [32, 33]. Thus, it acts against the glutamate excitotoxicity neuronal degeneration process, which constitutes one of the proposed molecular mechanisms of developing ALS [34]. Another recognized mechanism of action for riluzole is the noncompetitive antagonism of N-methyl-D-aspartate (NMDA) receptors [35] (Figure 1).

It is recommended that as soon as ALS patients with less than five years duration, no tracheostomy, and forced vital capacity (FVC) > 60% are diagnosed, they should be offered riluzole 50 mg twice a day [30, 36, 37]. In one of the systematic reviews that included three placebo-controlled randomized clinical trials with 876 riluzole and 406 placebo-treated patients, riluzole, however, demonstrated only two months' survival benefit over the control group [36]. These trials have shown that riluzole improves the rate to achieve at least one more year survival by 15% more than the placebo, as well as improving survival by three months compared to the placebo after 18 months of treatment [30]. You may need to treat eleven patients in order to delay one death by 12 months compared to the placebo [30]. However, these trials were criticized for not including patients at early stage of the disease. When five different clinical databases were analyzed retrospectively, riluzole was shown to improve survival by 6–21 months [30, 38]. It was also shown to delay progression from a mild-to-moderate disability state to a severe disability state [39]. Riluzole requires monitoring for liver enzymes, which is often done every month for three months, then every three months for another nine months, and then once per year thereafter. Other reported adverse effects of riluzole are very minimal and of no major concern [36].

### 3.2. Edaravone

Edaravone was used originally in patients with acute ischemic stroke to improve functional neurological impairments [40, 41]. It is a potent free-radical scavenger that interferes with the oxidative stress associated with ALS [42] (Figure 1).

Early clinical phases of testing the drug on animals have shown promising results in suppressing the denervation atrophy and degeneration of motor neurons in rodent ALS-like models [43, 44]. The results of an open-label phase II nonrandomized clinical trial showed that the rate of decline in the Revised ALS Functional Rating Scale (ALSFRS-R) score was 2.3 points during the six-month treatment period compared to 4.7 points over the six months prior to the treatment (*p* = 0.036) [45]. A large-scale phase III confirmatory clinical trial was conducted in Japan [46] which included 101 and 104 edaravone and placebo-treated patients, respectively. Although the edaravone group had smaller decline (change in ALSFRS-R scores: −5.70 [SE 0.85] versus −6.35 [SE 0.84]), the difference was statistically insignificant (*p* = 0.411). However, a post hoc analysis has shown that, in a restricted subgroup of the study, edaravone was effective in delaying the progression of the disease [46]. Another phase III double-blind placebo-controlled study was conducted where edaravone and placebo were administered to 68 and 66 patients, respectively [47]. This study restricted enrollment to only patients who met criteria for the subgroup analysis, i.e., who benefited from edaravone in the post hoc analysis of the previous trial. The recruitment criteria included the following: a definitive or probable ALS diagnosis according to the revised El Escorial criteria [48], ages 20–75 years, grade 1 or 2 ALS according to the Japan ALS Severity Classification, scores of 2 or more points on all items of ALSFRS-R, FVC ≥ 80%, and a maximum of two years of disease duration from the first symptom. The results supported the efficacy of edaravone (change in ALSFRS-R scores, from baseline to week 24 after treatment: −5.01 [standard deviation (SD): 0.64] with edaravone versus −7.50 [SD: 0.66] with the placebo) (*p* = 0.001). This means that edaravone slowed the disease progression by 33%, as measured by ALSFRS-R. Prior studies found that a 20% slower progression in ALSFRS-R is clinically meaningful. In other terms, edaravone may have saved the patients two months of progression for every six months of treatment.

These results, along with the lack of noted serious adverse effects, made edaravone the second agent to be approved by the FDA for ALS [47]. It is difficult, however, for clinical neurologists to prescribe edaravone for ALS for many reasons. The aforementioned criteria for the post hoc analysis are very stringent, such that it is expected that only 7% of ALS patients are eligible for edaravone. The last phase III clinical trial was run through only 24 weeks; thus, the long-term functional effects, side effects, and overall survival data are not yet available [49].

### 3.3. Masitinib

Masitinib is one of the tyrosine kinase inhibitors that modulate the neuroinflammation associated with many neurodegenerative disorders [50]. An experiment was run on SOD1^G93A^ rat progeny where treatment with masitinib commenced following paralysis onset. It showed that masitinib exerted neuroprotection by controlling microgliosis and neuroinflammation and ameliorating the aberrant glial cell proliferation in SOD1^G93A^ rats [51]. The results of the study were encouraging since the survival in postparalysis SOD1^G93A^ rats was prolonged significantly; there was a 40% prolongation when administered from days 1 to 7 to the postparalysis group compared with controls, with survival periods of 30 and 20 days, respectively (*p* < 0.01) [51]. Masitinib is unique among other tested agents in that its results were appealing when administered following disease onset.

A phase III clinical trial was completed on masitinib added to riluzole, and we are awaiting the full results to be revealed (Clinicaltrial.gov NCT02588677). Patients have been recruited for the trial with the following inclusion criteria: a duration of disease onset less than 3 years and FVC > 60%. In contrast to the trials of edaravone, the inclusion criteria in the undergoing phase III trial for masitinib are broader such that it encompassed a wider subgroup than the edaravone trials did [51, 52]. The masitinib trial included normal progressors (ALSFRS-R < 1.1 drops per month) and fast progressors (ALSFRS-R > 1.1 drops per month). Preliminary analysis showed benefits in the normal progressors by slowing the disease progression by 27%, but the benefit was not evident in the fast progressor group.

## 4. Gene and Cell Therapy

Considering the failure of neuroprotective agents over the past two decades to provide a cure, advancements in gene and cell-based therapeutic approaches should be discussed. Gene therapy is concerned with correcting faulty genes by means of delivering external genetic material and manipulating gene expression. The toxic gain of function is the mechanism thought to lead to FALS caused by SOD1 mutation. The mutant SOD1 gene produces a toxic protein that leads to motor neuron death. The proposed genetic therapy relies on introducing RNA material that targets the SOD1 gene as well as silencing its expression [6]. Preclinical trials using viral vectors encoding for RNAi (interference RNA) injected in SOD1 mice models resulted in reduced SOD1 protein levels and extended survival among the transgenic mice [53–55]. No data on humans are available yet. Another RNA-based technology is the antisense oligonucleotide (ASO) which binds to pre-mRNA and regulates the gene expression [56–59]. However, the phase I trial did not show that this method was effective [56]. The ASO has been studied to control the expression of the other common mutation linked to ALS C9ORF. However, success in this regard is neither consistent nor clinically significant yet [56]. Similarly, clinical trials have not identified definite benefits from an intramuscular injection of a plasmid that encodes for hepatocyte growth factor or encodes a gene to upregulate vascular endothelial growth factors [56].

## 5. Stem Cell Transplantations

Mesenchymal stem cells (MSC) can induce the release of growth factors and enhance the regeneration of degenerated neurons. Moreover, they act to reduce the implicated inflammation, support the motor neuron, and repair damaged cells [60].

Glass et al. conducted phase I and II clinical trials in the injection of human spinal cord-derived neural progenitor cells (NPC) [61, 62]. In the second trial [62], they included 15 ALS patients who were injected with human spinal cord-derived neural stem cells (HSSCs). Two cases had severe surgical complications (central pain syndrome; spinal cord edema and paraparesis). Four other cases had some side effects attributed to the immunosuppressants administered before the injections. The progression of the disease by means of ALSFRS-R, FVC, and grip strength was also assessed by comparison with three historical control groups. The slopes of decline demonstrated no difference in progression rates. The spinal cord transplantation of human stem cells was regarded as Class IV evidence for patients with ALS as a safe procedure that does not lead to the disease worsening. However, the study was not designed to test for the efficacy in slowing the progression of the disease. Larger scale studies are required before reaching such a conclusion [62].

A consensus regarding designing relevant human stem cell trials has been addressed [63].

## 6. Role of Diet, Weight Stabilization, and Enteral Feeding on Survival

Hypermetabolism, upper extremity weakness, and dysphagia collectively make ALS patients prone to weight loss, which may occur in 56% of ALS patients [64, 65]. It is known that more than 80% of ALS patients will sustain dysphagia; however, up to 39% of ALS patients may experience weight loss without having dysphagia [64, 66]. It is estimated that 50% of ALS patients suffer malnutrition [67]. Several studies demonstrated that >5% weight loss at the time of diagnosis predicts shorter survival and poor prognosis, with a 30% increase in the risk of death [67, 68]. Moreover, consistent results have been shown regarding higher body mass index (BMIs) (30 to 35 kg/m^2^) and its association with extended survival [69].

There are four known studies that have studied the effect of different diets in ALS [70–72]. A phase II clinical trial with 24 ALS patients has shown that the hypercaloric high carbohydrate diet group had a longer survival than the placebo group (0% death versus 43% death, respectively, over five months of observation) [70]. Moreover, the ALSFRS-R scores for these patients declined more slowly (−1.07 points per month) than the control group (−2.17 points per month). Despite that, this was not statistically significant when compared with the control group (*p* = 0.07) [70]. Another prospective noncontrolled study found that a high caloric diet (either high fat or high carbohydrate) results in weight stabilization but does not preclude progression in ALSFRS-R [72]. A third prospective controlled study investigated the effect of a high protein diet and showed that it may stabilize ALSFRS-R (i.e., a 2.1 point drop in ALSFRS-R in the high protein diet group compared to a 3.4-point drop in the control group over four months) and increases weight [71]. A recent systematic review proposed that further evidence is required before conclusive determination of whether a high fat or high carbohydrate diet is preferred with regard to survival prolongation; however, it is almost clear that unmanaged weight loss in ALS patients is associated with worse prognosis [73].

The enteral nutrition concept through percutaneous endoscopic gastrotomy (PEG) feeding is a guideline-recommended intervention [30, 37]. PEG feeding was shown to stabilize weight compared to the group who refused it [37]. The studies that included an appropriate control group have demonstrated a survival advantage in the group using PEG feeding [37, 74, 75]. Enteral feeding should be discussed with the patient once dysphagia or malnutrition (loss of weight exceeding 10%) ensues. A postmortem analysis on 80 ALS patients for discerning causes of death found that both noninvasive ventilation (NIV) and PEG exhibited significant survival benefit (i.e., 40 months with PEG versus 30 months, and more pronounced benefits in the limb onset of the disease) (*p* < 0.01) [76]. On the other hand, there are two recent respective studies that might suggest a more harmful effect of PEG feeding on both survival and disease progression, with a possible 48% decline in survival time [77, 78]. There are two variables that might be partly responsible for the contradicting data regarding the effect of a PEG tube on ALS. These factors are bulbar weakness and respiratory weakness. Respiratory weakness causes weight loss by itself, and it is likely possible that PEG feeding will benefit only patients with good respiratory muscle function and have no benefits among patients who have respiratory weakness [78].

Another controversial subject is whether or not PEG should be placed in patients with FVC < 50%. The American Academy of Neurology (AAN) guidelines classify patients into low, moderate, and high risk based on FVC values >50%, 30–50%, and < 30%, respectively [37]. This risk grading was also consistent with the study that analyzed the effect of PEG on survival from data extracted from a recent randomized clinical trial [78]. However, it was shown that using noninvasive ventilation during the PEG insertion procedure makes the procedure relatively safe for patients with respiratory weakness [79]. If PEG is deemed a risky intervention, percutaneous radiologic gastrostomy (PRG) can be used as a favorable alternative. If both PEG and PRG are not suitable, nasogastric tube feeding is recommended [30, 37]. PEG, PRG, and per-oral image-guided gastrostomy were all found to be equally similar with regard to safety and survival benefits [80].

## 7. Antioxidant Supplements

Oxidative stress is thought to be a constitutive component of the ALS pathogenesis [81]. Therefore, it is reasonable to hypothesize that antioxidants can manipulate the course of ALS and prolong survival (Figure 1).

### 7.1. Vitamin E

Vitamin E underwent several studies to investigate its role as an antioxidant in slowing ALS progression. There are two placebo-controlled clinical trials that failed to show survival benefits from vitamin E [82, 83]. However, there was a slowing of disease progression in one of these two studies during the 12-month period; 32% of patients on vitamin E progressed from mild to moderate state compared to 44% in the placebo group [83]. In a large epidemiological dataset (1 million participants, with 805 participants developing ALS), it was found that taking vitamin E may reduce the risk of developing ALS, particularly with a longer duration of use [84]. However, further investigation is necessary to clarify its impact on survival [82, 83, 85–87]. In clinical practice, some experts recommend using 400 units of vitamin E per day for ALS patients.

### 7.2. Vitamin D

There are multiple papers that support the concept of better prognosis with vitamin D supplementation [88–93]. One of the findings that led to this concept is that ALS patients usually have low vitamin D levels at the time of diagnosis [88, 94]. Several mechanisms have been proposed, including increasing the level of neurotrophic factors such as insulin-like growth factor-1 (IGF-1) and vascular endothelial cell growth factor (VEGF) and increasing the calcium binding protein which reduces the damage caused by calcium influx [90]. Vitamin D may also reduce oxidative stress by reducing tumor necrosis factor alpha (TNF-a), interleukin 1 beta (IL-1b), and nitric oxide synthase (NOS) [89]. A retrospective study of a small cohort (74 patients with documented vitamin D levels) found a shorter median survival with low vitamin D levels at the time of diagnosis (52.8 months in a group with normal vitamin D levels versus 29.5 months in the group with severe vitamin D deficiency) [88]. Karam et al. found that supplementation with 2000 IU of vitamin D daily slowed the progression of the disease over a nine-month period, as measured by ALSFRS-R, when compared to patients who did not receive vitamin D. The difference in the rate of ALSFRS-R decline was 1–4 points in favor of vitamin D, but this was not maintained at 12 months [94]. In contrast to the above, despite the fact Blasco et al. found a low mean vitamin D level among 125 ALS patients, they also found that higher vitamin D levels were associated with a worse ALSFRS-R score during the course of ALS disease [95]. Similarly, Yang et al. found no survival advantage among patients with higher mean vitamin D level [96]. The role of neuroprotection and survival benefit for vitamin D3, however, remains elusive, and there is no apparent correlation between vitamin D3 level and survival [95, 96]. Further investigation is warranted to establish whether it will yield significant outcomes to recommend dietary supplementation with vitamin D3.

### 7.3. Vitamin A

There have been no clinical trials for the efficacy of vitamin A in patients with ALS. However, vitamin A (alpha carotene, beta carotene, and retinol) levels in blood in 40 ALS patients were comparable to the levels in 87 normal controls [97]. It was shown in a mice study that it may shorten the lifespan in an ALS mice model [98]. Despite that, it is common practice that ALS specialists prescribe 25,000 units daily of vitamin A as part of the antioxidant regimen.

### 7.4. Vitamin C

A pooled analysis of five different studies failed to demonstrate a reduced risk of developing ALS with vitamin C intake [99]. However, there is no clinical data on the efficacy of vitamin C to slow disease progression. Despite that, it is common practice that ALS specialists prescribe 1 gram of vitamin C three times daily as part of the antioxidant regimen.

## 8. Role of Multidisciplinary Management

Because of the multiple impairments and disabilities sustained by ALS patients, multidisciplinary clinics specialized in ALS (MDCs) have emerged and have shown compelling evidence of improving quality of life and lengthening survival compared to the general neurology clinics [30, 100, 101]. The rationale is to increase patient accessibility to the needed resources with optimal expertise in this disease. This easy accessibility is important particularly given that the pace of changing health needs for ALS patients is faster than in all other neurodegenerative diseases. MDCs will lead to coordinated care and better quality of life and survival advantage. James Rooney et al. found a survival advantage among 340 patients who attended MDCs when compared to 169 patients who attended general neurology clinics (Hazard Ration (HR) 0.59, 95% CI = 0.49–0.71,* p* < 0.001) [102]. Moreover, this statistically significant difference was echoed when the multivariate analysis was performed. Thus, attending an MDC was an independent predictor of survival. Similarly, Traynor et al. found that 74 ALS patients who attended the MDCs had an average of 7.5 months' survival advantage compared to 262 patients who attended general neurology clinics [100].

MDCs are more cost-effective than nonspecialized clinic as they reduce the rate of hospitalization. The rate of hospitalization was 1.2%, mostly for planned intervention, with a median stay of six days if an MDC was attended. In comparison, the rate was 3.3% if a general neurology clinic was attended, with a median hospital stay of 13 days [103]. MDCs comprehensively manage complex issues found in ALS. For example, MDCs can assist in managing the bulbar symptoms (e.g., dysphagia, respiratory compromise, and dysarthria), psychosocial problems, and nutritional deficiencies. The European Federation of neurological sciences (EFNS) and the AAN recommend that the MDCs should be available as soon as possible for patients, as well as for the family caregivers, to achieve optimal health care. The AAN gave MDCs a level B recommendation for survival advantage and level C recommendation for the quality of life [30, 37, 104]. MDCs should ideally be composed of a neurologist with ALS expertise, as well as many other physicians involved in care during the progression of the disease.  Table 2 lists the comprehensive components of MDCs for optimal care to be provided to ALS patients [105].

Palliative care is also an underpinning part of multidisciplinary management which has been shown to improve quality of life and is appropriate to start once the diagnosis of ALS is made [30, 106]. Palliative care ensures that the symptoms of the patients are appropriately managed. Moreover, the palliative care team should be involved in discussing critical decisions with the patients, such as the risks and benefits of ventilatory interventions in patients with respiratory compromise [106].

## 9. Noninvasive Ventilation

Respiratory failure, with or without pneumonia, is considered the leading cause of death in ALS [107]. It results when the motor neurons supplying the respiratory muscles (diaphragm and intercostal muscles) degenerate progressively [1, 30]. The associated signs and symptoms include dyspnea on minimal exertion, orthopnea, decreased chest movement, morning headache, and confusion (due to CO2 retention) [108, 109]. Increased secretions—another bulbar symptom due to dysphagia—also possibly causes aspiration pneumonia. Respiratory functioning, thus, needs to be monitored regularly to detect and manage respiratory muscle weakness in a timely manner, before serious complications occur.

FVC, maximum inspiratory pressure (MIP), and sniff nasal inspiratory pressure (SNP) are widely used to assess for respiratory function deterioration among ALS patients.

The NIV should be considered as soon as vital capacity declines below 50%, MIP becomes <−60 cm, or the SNP becomes <40 cm H2O [37, 110]. A randomized clinical trial on 92 patients showed a survival benefit of 206 days for patients on NIV compared to standard care. The benefits were more evident in patients with mild and moderate bulbar dysfunction compared with severe bulbar dysfunction. Quality of life, however, was improved in both groups [111]. A retrospective study of 122 patients found that survival was 14 months in bilevel positive airway pressure (BiPAP) patients, compared to 5–7 months for patients not using BiPAP. Also, they found that the rate of decline in FVC was less among patients on BiPAP (−3.5/month if using BiPAP versus −6/month otherwise) [112]. The NIV should be used >4 hours per day to gain the survival advantage. A recent Cochrane review concluded that there is a moderate quality of evidence to support the survival benefits from NIV [113]. The NIV is recommended in both AAN guidelines (as level B for survival benefits and level C for quality of life and slow respiratory decline) and in EFNS guidelines as a therapeutic approach to respiratory insufficiency [30, 37].

## 10. Invasive Ventilation

Invasive ventilation (IV) is another option that may prolong survival for 10 to 30 years [114]. Tagami et al. reviewed survival data from 160 patients in Japan, of whom 52 patients were on tracheostomy ventilation, and found significant survival benefits in the IV group (74 months) compared to the NIV (48 months) and no ventilation group (32 months) [115]. Another retrospective review from Italy found significant survival benefits from tracheostomy invasive ventilation (TIV), with 47 months of survival benefits in the TIV group compared to 31 months in the no tracheostomy group [116]. Another retrospective study reviewed the outcome of 60 patients who presented to the hospital with acute respiratory failure and required intubation. 70% became completely dependent on TIV, 28% partially dependent on TIV, and 1.6% (one patient) was independent of IV. The median survival after TIV was 21 months (0–155 months); 65% were alive at 1 year and 45% were alive at 2 years after discharge, with the most common cause of death being pneumonia (46%). In this study, they interviewed 13 patients and 11 expressed that they were willing to undergo a tracheostomy if they had to make the decision again. Moreover, 15% of the TIV group were severely depressed [117]. Dreyer et al. reviewed the survival in 431 ALS patients and found that the mean survival among patients who did not receive mechanical ventilation was 23 months (146 patients) versus 26 months for NIV (173 patients), versus 57 months for NIV followed by IV (69 patients), and versus 34 months for IV alone (21 patients) [118].

Chio et al. reported a median survival of 253 days (<1 year) after tracheostomy among 134 ALS patients, while Sancho et al. reported that 78% of 38 tracheostomy patients had a one-year survival [119, 120]. The major cause of death in TIV was found to be respiratory infections (46%) followed by sepsis (31%) [121]. There are few reported cases of survival >20 years after tracheostomy placement [122]. Patients on TIV are able to live many years until pneumonia develops or sudden death occurs due to hypotension and circulatory collapse [123].

There are few major downsides that may affect the patient's decision to proceed with TIV or not. The probable major limitation is the inability to communicate when the disease is advanced, as patients lose ocular movements. Nakayama et al. retrospectively reviewed 76 ALS patients on TIV and found that 17–25% of them have no means to communicate (also known as totally locked-in state or TLS) [124]. Hayashi et al. also retrospectively reviewed the rate of TLS among 70 ALS patients with TIV and found this to be 11.4%. If the patient lived more than 5 years it became 18.2%, while 33% had very limited communication [125].

The decision to use TIV should be balanced with the available social supports that are needed to maintain quality of life. Many ALS expert neurologists believe that most ALS patients will decide not to pursue TIV if they planned in advance and if they were aware that they will become a burden on their family or have no means of communication [126].

## Figures and Tables

**Table 1:** Interventions that may lead to survival benefits in ALS patients.

Intervention	Mechanism	Possible survival and progression benefits
Riluzole	Glutamate antagonist	3 months (possibility > 6 months if used early on)

Edaravone	free-radical scavenger that interferes with the oxidative stress	Slows disease progression by 33% in selected subgroup of ALS patients

Masitinib	Tyrosine kinase inhibitor. Modulates neuroinflammation and microgliosis	Slows disease progression by 27%

Gene therapy	Antisense oligonucleotide Silences SOD1 expression	No available data

Stem cell transplantation	Release of growth factors and enhance regeneration of degenerated neurons	No efficacy was demonstrated in phase II studies

Vitamin E	Antioxidant	Reduces the risk of developing ALS Conflicting data on efficacy on disease progression

Vitamin D	Antioxidant Increase neurotrophic factors Increase calcium binding protein	Slows the disease progression by 1–4 points on the ALSFRS-R^*∗*^

Multidisciplinary clinic	Early institution of interventions with survival benefits Monitoring and preventing complications Reduces hospitalization duration	7.5 months

Enteral feeding	Weight stabilization	10 months but recent data argue against this

Non-invasive ventilation	Improves oxygenation by supporting respiratory muscle function Slows rate of FVC^#^ decline	5–7 months

Invasive ventilation	Improves oxygenation by replacing respiratory muscle function	1–2 years

^*∗*^ALSFRS-R: amyotrophic lateral sclerosis functional rating scale-revised.

^#^FVC: forced vital capacity.

**Table 2:** Comprehensive components of the multidisciplinary caring clinics for amyotrophic lateral sclerosis patients.

**Health professional** **∗**	**Issues involved in caring for**	**Intervention**
A neurologist with expertise in ALS	General care of the patient disease and progression	Riluzole and edaravone, Botox© injection for sialorrhea, spasticity, medication prescription

Specialized ALS nurse	Dysphagia, respiratory issues, general self-care	Coordinating the care provided by other health professionals in various interventions

Pulmonologist/respiratory therapist	Respiratory issues related to respiratory muscle weakness	BiPAP, Assisted ventilation techniques (e.g., NIV)

Dietitian, swallow therapist	Dysphagia, sialorrhea, weight stabilization, nutritional deficiencies	Providing general advice on nutritional habits, recommending PEG

Speech pathologist	Communication compromise (e.g., dysarthria)	Providing assistant communication devices

Physiotherapist	Limb weakness	Mobility support by appropriate equipment. Recommend appropriate home modifications

Occupational therapist	Hand weakness	Evaluating for appropriate equipment

Neuropsychologist/psychologist, palliative care physician	Identifying concerns of the patient and relatives	Providing psychological support and counseling, caring for the grieving family

ALS: amyotrophic lateral sclerosis; NIV: noninvasive ventilation; PEG: percutaneous endoscopic gastrostomy.

*∗*ALS and motor neuron disease national/international associations should also engage in providing support for the patient.
